# Intact mitochondrial substrate efflux is essential for prevention of tubular injury in a sex-dependent manner

**DOI:** 10.1172/jci.insight.150696

**Published:** 2022-04-08

**Authors:** Allison McCrimmon, Kerin M. Cahill, Claudia Kruger, Margaret E. Mangelli, Emily Bouffard, Timothy Dobroski, Kelly N. Michanczyk, Susan J. Burke, Robert C. Noland, Daria V. Ilatovskaya, Krisztian Stadler

**Affiliations:** 1 Oxidative Stress and Disease Laboratory,; 2 Immunogenetics Laboratory, and; 3 Skeletal Muscle Metabolism Laboratory, Pennington Biomedical Research Center, Baton Rouge, Louisiana, USA.; 4 Department of Physiology, Medical College of Georgia, Augusta University, Augusta, Georgia, USA.

**Keywords:** Metabolism, Nephrology, Chronic kidney disease, Fatty acid oxidation, Mitochondria

## Abstract

The importance of healthy mitochondrial function is implicated in the prevention of chronic kidney disease (CKD) and diabetic kidney disease (DKD). Sex differences also play important roles in DKD. Our previous studies revealed that mitochondrial substrate overload (modeled by homozygous deletion of carnitine acetyl-transferase [CrAT]) in proximal tubules causes renal injury. Here, we demonstrate the importance of intact mitochondrial substrate efflux by titrating the amount of overload through the generation of a heterozygous CrAT-KO model (PT-CrAT^HET^ mouse). Intriguingly, these animals developed renal injury similarly to their homozygous counterparts. Mitochondria were structurally and functionally impaired in both sexes. Transcriptomic analyses, however, revealed striking sex differences. Male mice shut down fatty acid oxidation and several other metabolism-related pathways. Female mice had a significantly weaker transcriptional response in metabolism, but activation of inflammatory pathways was prominent. Proximal tubular cells from PT-CrAT^HET^ mice of both sexes exhibited a shift toward a more glycolytic phenotype, but female mice were still able to oxidize fatty acid–based substrates. Our results demonstrate that maintaining mitochondrial substrate metabolism balance is crucial to satisfying proximal tubular energy demand. Our findings have potentially broad implications, as both the glycolytic shift and the sexual dimorphisms discovered herein offer potentially new modalities for future interventions for treating kidney disease.

## Graphical abstract

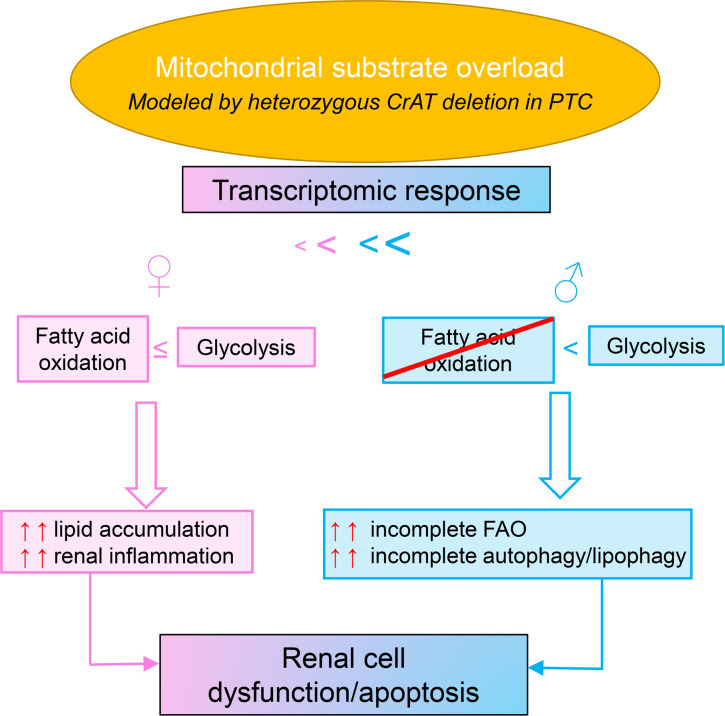

## Introduction

Chronic kidney disease (CKD) and metabolic disease or diabetic kidney disease/nephropathy (DKD/DN) are of major health concern (1, 2). Maintenance of intact mitochondrial function is crucial for the prevention of CKDs, especially those resulting from metabolic complications (3–8). Mitochondrial dysfunction, however, can be defined in many different ways. As such, it is not entirely clear in what way mitochondrial dysfunction impacts kidney disease development. This is an especially critical question for proximal tubular cells (PTC), as they have a high energy demand, with heavy reliance on mitochondria and fatty acid oxidation (FAO) (9). These cells are not equipped with much glycolytic capacity (10, 11) to compensate for a significant loss of mitochondrial function. Therefore, impairments in FAO and/or mitochondrial substrate metabolism can rapidly affect proximal tubular cell function, eventually leading to apoptosis and cell death (12–16). Importantly, PTC loss is a very reliable predictor of end-stage renal disease and mortality in patients (17).

Mitochondrial substrate overload is a phenomenon that occurs under states of chronic overnutrition, in obesity, and in type 2 diabetes in mice and humans (18, 19). It was first described in relation to skeletal muscle substrate switching, in the context of insulin resistance (20). As a result of overnutrition, mitochondria are presented with excessive amounts of substrates, but this is not necessarily matched with the energy need and ATP demand. Overburdened mitochondria show high rates of incomplete FAO (21, 22), accumulation of excess ac(et)yl-CoA moieties, a change in NADH/NAD^+^ levels, consequent redox imbalance/oxidative stress, and allosteric inhibition of several metabolic enzymes (20, 23). To date, mitochondrial substrate overload has largely been studied in the skeletal muscle, and the consequences of a potential mitochondrial overload in CKD are relatively unknown.

In our previous work, we discovered that mitochondrial substrate overload itself causes tubular and glomerular injury in male mice (24). To model the conditions occurring during mitochondrial overload, we created mice lacking the enzyme carnitine acetyl-transferase (CrAT) in the PTC. CrAT is a crucial enzyme residing in the mitochondrial matrix, serving as a “relief valve” for acetyl-CoA. A major role of CrAT is to link excess acetyl-CoA and mostly short-chain acyl-CoA metabolites to carnitine, thereby converting them to membrane permeable products and allowing transport from the mitochondria, while liberating free CoA within the mitochondrial matrix (21, 22). Deletion of CrAT, therefore, models a scenario of substrate excess where export of carbons is limited — much like a scenario that can happen in obesity and type 2 diabetes. Mice with PTC-specific CrAT deletion develop tubular disease and secondary glomerular injury (24), recapitulating many features of DKD but with fewer confounding factors. Mice with mitochondrial substrate overload in the PTC exhibited increased serum creatinine levels, albuminuria, tubular atrophy, and secondary glomerulosclerosis, and the kidneys had increased levels of partially oxidized products from incomplete FAO. To our knowledge, this is the first model that allows for exclusively isolating and studying the biological aspects of mitochondrial overload in PTC — a phenomenon exhibited by human diabetic kidneys (25).

Based on our previous results, we asked whether having only 1 allele of CrAT is sufficient to fully or at least partially restore mitochondrial substrate imbalance and alleviate renal damage. We also examined disease development in female mice, given the importance of potential sex differences in renal disease (26–29). Sex-related differences have a significant impact on the development of microvascular complications in diabetes (30). There is a growing body of literature suggesting that females are protected from several forms of kidney disease in many animal models and humans. The protective effect, however, seems to be diminished in the setting of diabetes (28). Significant sex differences were discovered recently in relation to CKD models, where CKD progressed faster in males (31). Differences in hemodynamic function between men and women with type 2 diabetes were also identified, with men having higher markers of hyperfiltration (32). Thus, we asked whether similar observations can be found in the mitochondrial overload model. Contrary to our initial hypothesis, at around age 12–15 months, heterozygous CrAT-KO mice (hereafter referred to as PT-CrAT^HET^ mice) fed a standard rodent chow also developed tubular injury, secondary glomerular damage, and significant changes in both mitochondrial structure and function, indicating the importance of a fully intact renal PTC mitochondrial carbon trafficking. We also demonstrate that mitochondrial overload engages vastly different renal transcriptional pathways in male versus female mice. Finally, we show that a glycolytic shift occurs in PTC in response to mitochondrial overload conditions. Taken together, our data emphasize the importance of an intact mitochondrial substrate balance in the PTC for the prevention of renal damage, and they highlight the significantly different responses and mechanisms that can occur due to sexual dimorphisms.

## Results

### PT-CrAT^HET^ male and female mice both develop tubular injury and secondary glomerulosclerosis.

**Figure 1:**
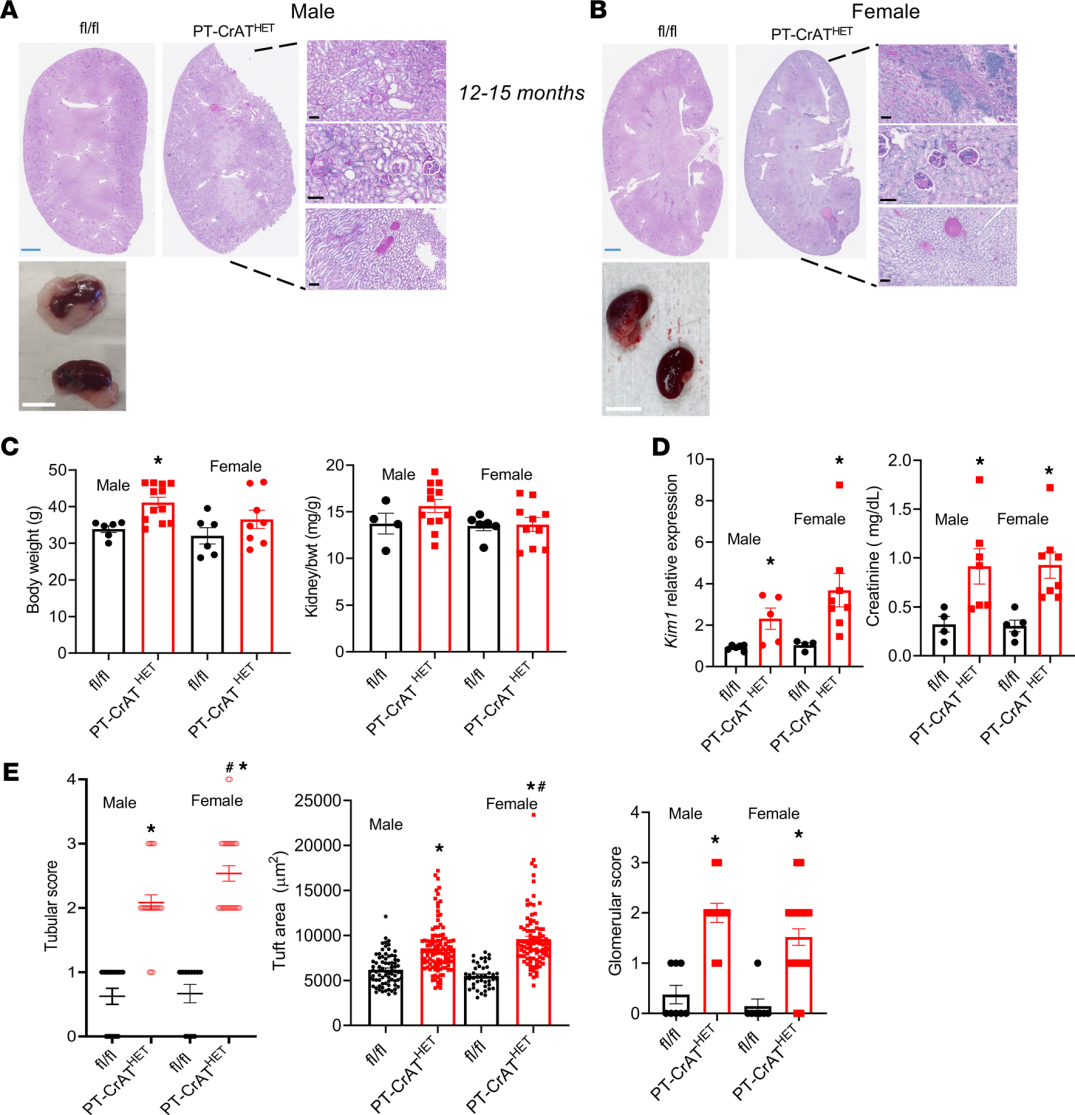
Impaired mitochondrial substrate efflux in PTC modeled by CrAT haploinsufficiency causes renal injury. (**A** and **B**) PAS staining of PT-CrAT^HET^ kidneys of male and female mice showing typical features of kidney injury when compared with fl/fl controls. Scale bars: blue, 2.5 mm; white, 10 mm; black, 100 μm. (**C**) Body weights and kidney/body weight ratios. (**D**) *Havcr1* (*Kim-1*) expression levels and serum creatinine levels. (**E**) Tubular injury scores, glomerular tuft area, and glomerular scores were analyzed and compared. *n* = 5–7 mice/group, *n* = ~30 viewing areas/mouse kidney at the same magnification. *P < 0.05 versus control, ^#^*P* < 0.05 versus male. Data are shown as mean ± SEM; 2-way ANOVA, Bonferroni’s post hoc test or Kruskal-Wallis test.

Mice with PTC-specific CrAT haploinsufficiency were created by breeding mice with the homozygous CrAT deletion to WT (C57BL/6J) mice (Supplemental Figure 1, A–D; supplemental material available online with this article; https://doi.org/10.1172/jci.insight.150696DS1). We examined the kidneys every 3 months. Interestingly, PT-CrAT^HET^ mice — similar to their previously published homozygous counterparts (24) — developed significant renal injury by 12–15 months of age (Figure 1 and Supplemental Figures 2 and 3). Females and males showed many similarities and a few notable differences, as well. Males had more tubular dilation and atrophy (Figure 1A), while females had more inflammatory cell infiltration around tubules (Figure 1B). Kidneys from both sexes showed substantial secondary glomerular injury and protein casts in cortex and medulla (Figure 1, A and B). Female kidneys were much easier to dissect clean (Figure 1B), while males always had significant perirenal fat surrounding them (Figure 1A). This is in agreement with the result of males having a significant increase in body weight when compared with controls or females (Figure 1C). Expression of *Havcr1* (*Kim-1*) and serum creatinine levels were significantly higher in KOs but similar in both sexes (Figure 1D). When scores were analyzed and compared between sexes (Figure 1E), females had worse tubular scores and larger glomerular tuft areas. Collectively, these data indicate that mitochondrial overload modeled by CrAT haplosufficiency causes CKD in both males and females, but the manifestation of the disease is different in males versus females.

### PT-CrAT^HET^ mice show mitochondrial ultrastructural changes, increased lipid deposition, and autophagy.

**Figure 2:**
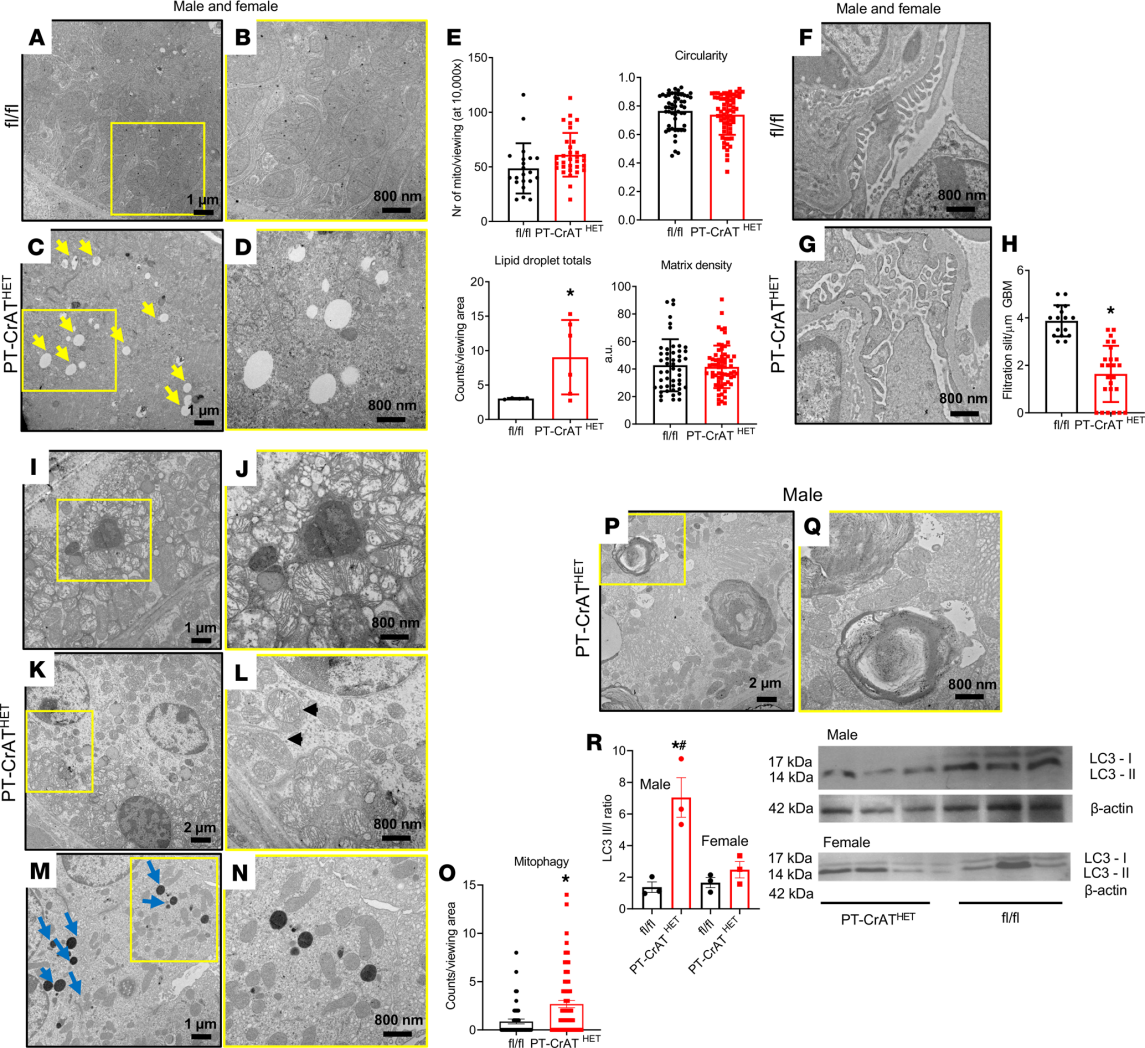
Ultrastructural analysis reveals lipid deposition and impaired autophagy in mitochondrial substrate overload–induced kidney disease. Representative TEM microphotographs at 10,000×, yellow squares indicate an image on the next subpanel at 20,000×. (**A**–**D**) Normal mitochondrial structure is shown in fl/fl mice (**A** and **B**) while PT-CrAT^HET^ mice display several large lipid droplets in PT cells (yellow arrows) (**C** and **D**). (**E**) Images were analyzed and mitochondrial number, circularity, and matrix density, and total number of lipid droplets were counted. (**F**) Normal podocyte foot processes in fl/fl mice. (**G** and **H**) Podocytes from PT-CrAT^HET^ mice have foot process effacement, analyzed as number of filtration slits/μm. (**I**–**N**) Further examples of ultrastructural damage seen in several of the PT-CrAT^HET^ samples are shown in representative photographs. Lipid droplets and mitochondria with fragmented cristae (**I** and **J**), mitochondria with double membranes indicating mitophagy (black arrowheads, **K** and **L**) autolysosome-like bodies (blue arrows, **M** and **N**; counted in **O**), and — in males only — several large multilamellar body-like structures (**P** and **Q**). (**R**) Western blot analysis of the autophagy marker LC3-I/II. All images were analyzed using ImageJ, *n* = ~30–40 pictures/mouse kidney at the same magnification. *P < 0.05. Data are shown as mean ± SEM; 2-tailed Student’s unpaired *t* test or Mann-Whitney *U* test.

To understand the effects of a substrate overload on the mitochondria, next we investigated changes in mitochondrial and cellular ultrastructure using a transmission electron microscope (TEM). Electron microscopy revealed normal mitochondrial morphology in the PTC (Figure 2, A and B) and normal foot process of podocytes in control littermates (Figure 2F). The number of mitochondria counted, their shape, and their matrix density were similar (Figure 2E). PT-CrAT^HET^ mice, however, had several medium- and large-sized lipid droplets accumulating in PTC (Figure 2, C and D) and foot process effacement in podocytes (Figure 2, G and H). The total number of lipid droplets/viewing area was significantly higher in the CrAT^HET^ deletion model (Figure 2E). Further detailed examination of the microphotographs from PT-CrAT^HET^ mice revealed mitochondria with disrupted and disorganized cristae, surrounded by lipid droplets (Figure 2, I and J); mitochondria surrounded by double membrane structures (Figure 2, K and L); and electron dense droplets and vacuoles, indicative of autolysosome activity (Figure 2, M–O). The most striking discovery was the presence of large multilamellar bodies (MLB) in males only, containing wrapped-up lamellar phospholipid membranes (Figure 2, P and Q). MLBs are typically formed when fusion of autolysosomes and autophagy/lipophagy are impaired. MLBs (also known as “zebra bodies”) have been shown to be present in large amounts in kidney cells in Fabry disease (33, 34). Next, impairments in autophagic processes in males were further confirmed, monitoring the ratio of the autophagy marker LC3-I/II (Figure 2R). These results collectively suggest that mitochondrial overload and blocking acetyl-CoA efflux induces significant structural injury in mitochondria, impacting cristae structure but not reducing their number or altering their shape. Mitochondrial overload also leads to the excessive accumulation of cellular lipid droplets in PTC. The formation of several large MLBs in males is likely indicative of a disrupted autophagy process and lipid storage disorder that was not observed in females.

### CrAT haploinsufficiency causes a glycolytic shift in PT.

**Figure 3:**
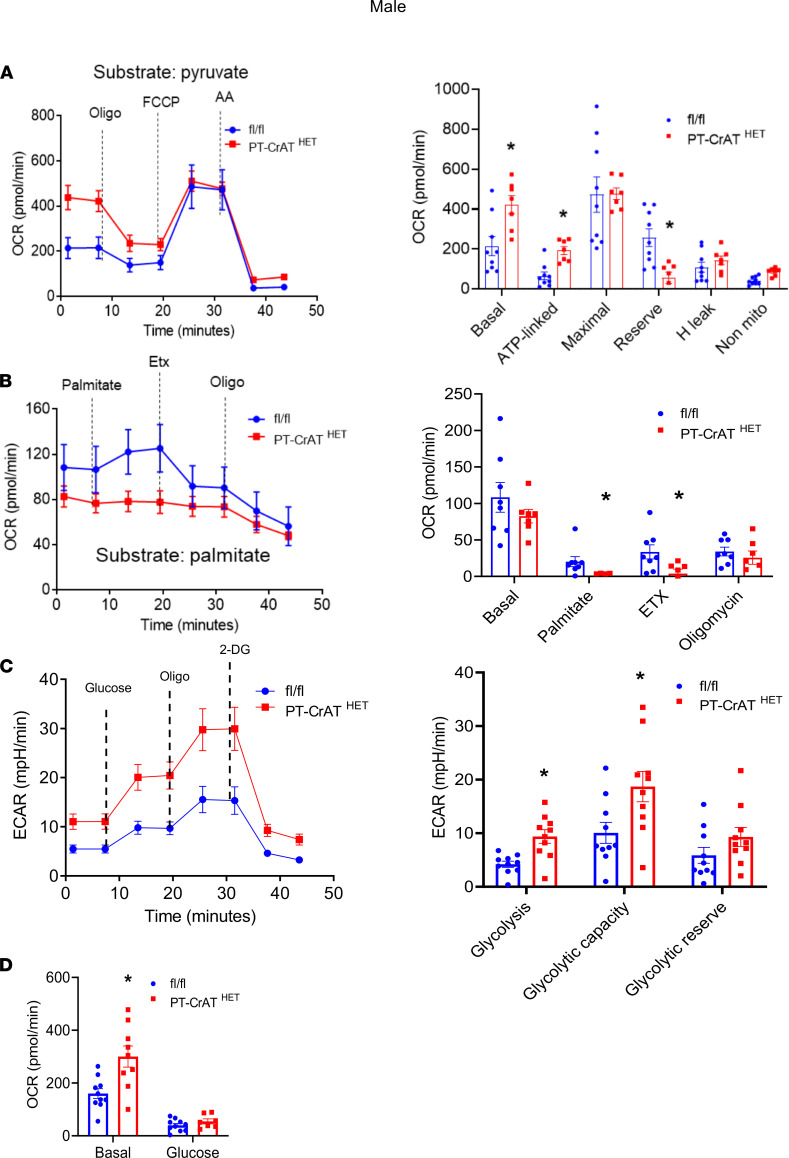
Glycolytic shift in PT-CrAT^HET^ tubules in males. Mitochondrial oxygen consumption rates (OCR) and extracellular acidification rates (ECAR) were measured in freshly isolated PT fragments from male mice. (**A**–**D**) Representative OCR/ECAR graphs showing a typical respiratory curve of PTC and its analysis using pyruvate (**A**), palmitate (**B**), or glucose (**C** and **D**). Oligo, oligomycin; AA, antimycin A; Etx, etomoxir; 2-DG, 2-deoxyglucose. *n* = 8 biological replicates, *n* = 10 technical replicates/experiment; 30 μg protein equivalent of PTC loaded/well. Data are shown as mean ± SEM. *P < 0.05; 2-tailed Student’s unpaired *t* test.

**Figure 4:**
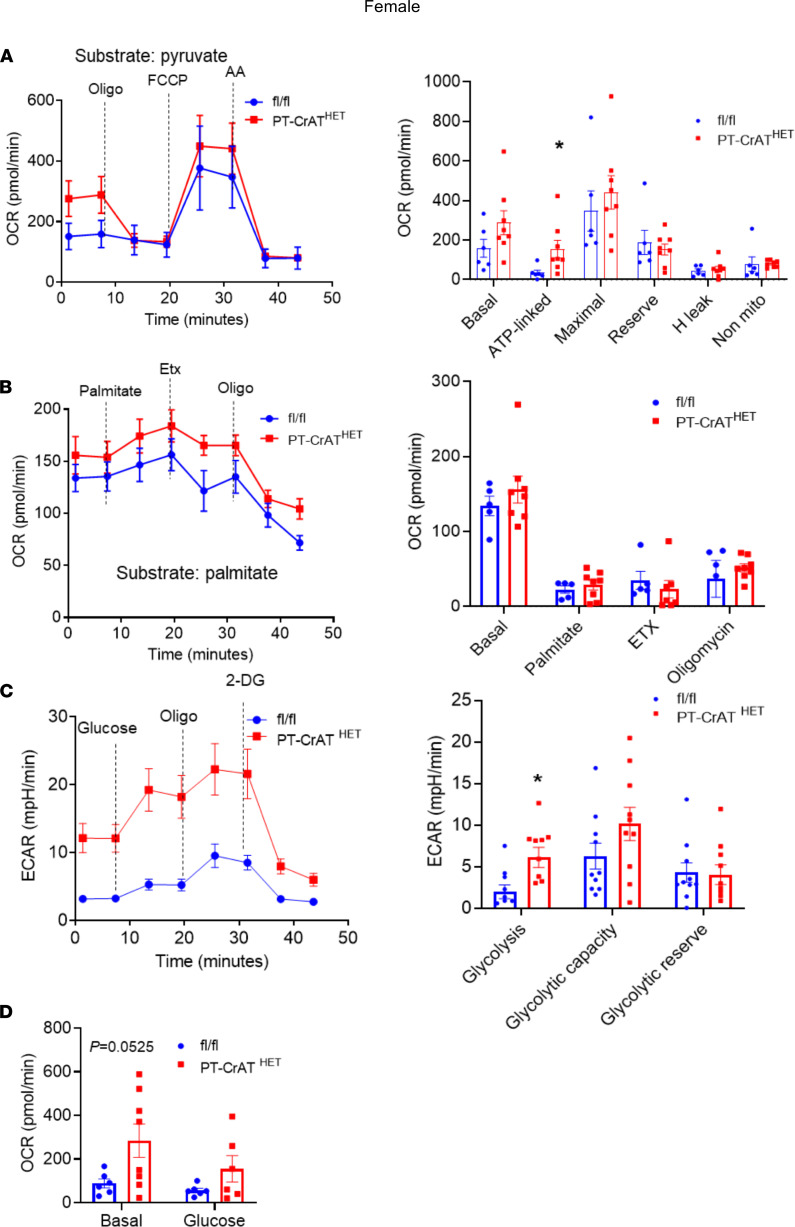
Glycolytic shift in PT-CrAT^HET^ tubules in females. Mitochondrial oxygen consumption rates (OCR) and extracellular acidification rates (ECAR) were measured in freshly isolated PT fragments from female mice. (**A**–**D**) Representative OCR/ECAR graphs showing a typical respiratory curve of PTC and its analysis using pyruvate (**A**), palmitate (**B**), or glucose (**C** and **D**). Oligo, oligomycin; AA, antimycin A; Etx, etomoxir; 2-DG, 2-deoxyglucose. *n* = 8 biological replicates, *n* = 10 technical replicates/experiment; 30 μg protein equivalent of PTC loaded/well. Data are shown as mean ± SEM. *P < 0.05; 2-tailed Student’s unpaired *t* test.

We reasoned that such significant changes of mitochondrial morphology and structure will be reflected in physiological and bioenergetic changes in PTC. Three experiments were designed to test this prediction using the Seahorse Extracellular Flux Analyzer. (a) To assess the ability of respiration on carbohydrate-based substrate, we measured oxygen consumption rates in freshly isolated PTC from the PT-CrAT^HET^ mice respiring on pyruvate. (b) OCR was measured after the addition of BSA-conjugated palmitate (FA-based substrate) and after the addition of etomoxir (ETX), which inhibits carnitine-palmitoyl transferase 1 (Cpt1), the rate limiting enzyme in (long chain) FAO transport to mitochondria. (c) PTC were supplemented with glucose, and extracellular acidification rates (ECAR) were monitored to test for a potential glycolytic shift. PTC nephron segments from both male and female heterozygous KO mice (12 months of age) displayed a significant (~2-fold) increase in basal respiration on pyruvate (Figure 3A and Figure 4A), which could be indicative of adaptation to the disease, some degree of uncoupling, or an increase in ATP-turnover. Consistently with the latter scenario, CrAT haplosufficient PTC also showed an increase in ATP-linked respiration. However, these cells had a marked decrease in reserve capacity, which is indicative of poor ETC integrity. PTC from females maintained their reserve capacity when compared with controls (Figure 4A). When palmitate was provided, PT-CrAT^HET^ males were unable to increase their OCR; females, however, were able to oxidize this substrate (Figure 3B and Figure 4B). We also found that PTC respirating on 10 mM glucose from both male and female PT-CrAT^HET^ mice significantly increased their glycolysis when compared with controls, and this is not a typical and natural energy source for PTC (Figure 3C and Figure 4C). Glycolytic capacity (after inhibiting ATP synthase with oligomycin) was only significantly increased in males (Figure 3C). When we monitored OCR under glycolytic conditions, basal respiration (similarly to the pyruvate-based experiments) again was increased in both sexes, but the addition of glucose did not induce a significant increase in mitochondrial OCR (Figure 3D and Figure 4D). These experiments altogether suggest that, when mitochondrial FAO pathways are significantly impaired in PTC, the cells attempt a glycolytic shift to provide some form of fuel for their energy needs. In light of the kidney injury phenotype, however, glycolysis does not seem to be sufficient to compensate for the loss of other, more efficient metabolic pathways. The bioenergetic data also suggest that such a glycolytic shift is much more prominent in male mice that suffered an almost complete inhibition of FA metabolism and were unable to use palmitate when compared with female mice, where some other means of substrate metabolism is likely preserved.

### Transcriptional changes and pathways contributing to kidney disease are different in male and female mice with mitochondrial overload.

**Figure 5:**
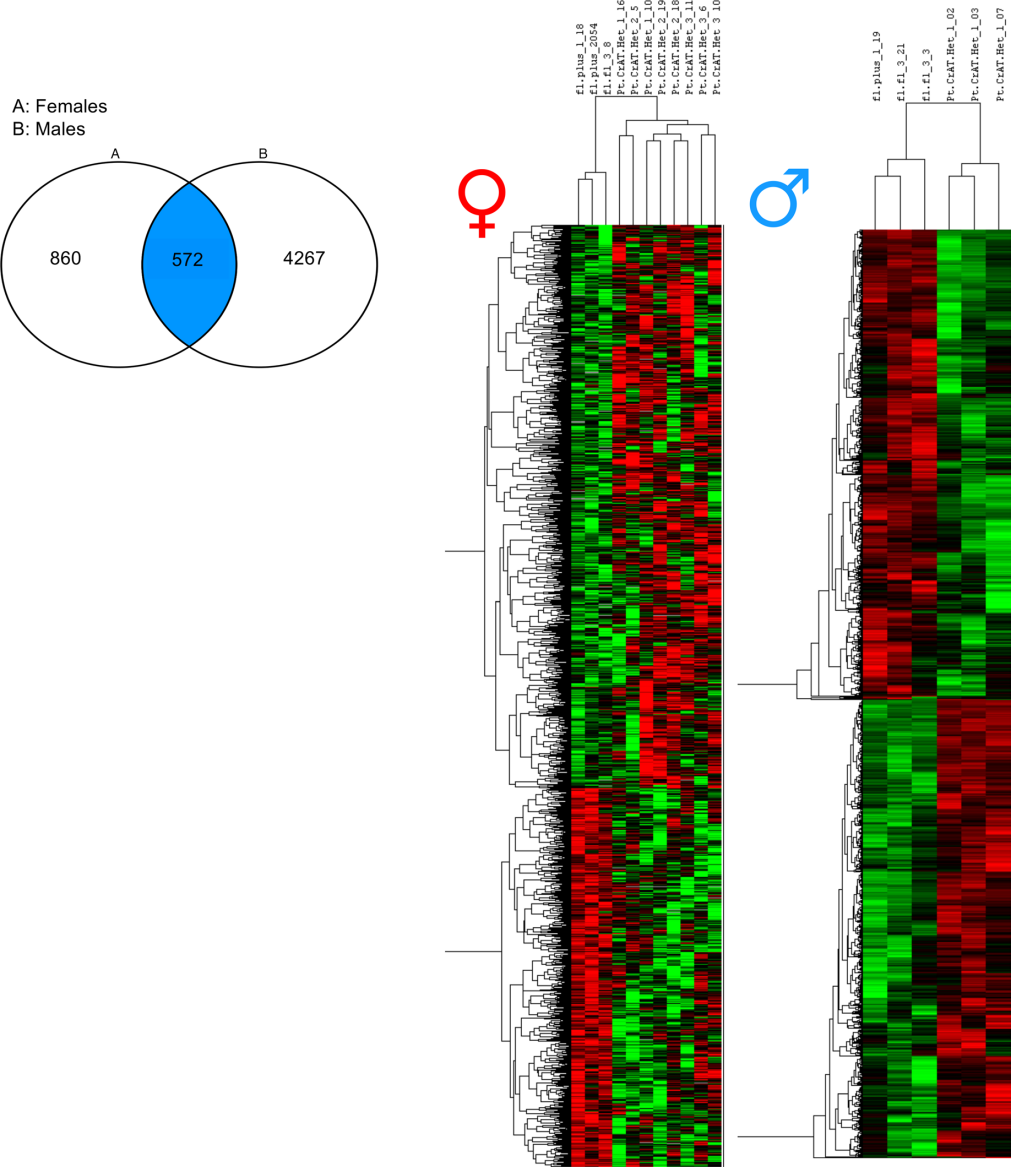
Impaired mitochondrial substrate efflux–induced transcriptomic responses in males and females. Transcriptomic analyses of male and female kidneys from PT-CrAT^HET^ mice were performed using NGS and Ingenuity Pathway Analysis. Left panel shows the number of differentially regulated/expressed genes in PT-CrAT^HET^ females versus males and genes that are common in both sexes. Right panel shows cluster analysis with the distribution of all up- versus downregulated genes and potential outliers in normal versus PT-CrAT^HET^ kidneys. *n* = 3–6 animals/group; genes with a *P* < 0.1 cutoff in differential expression were considered in the analysis.

To investigate and better understand the changes induced by acetyl-CoA imbalance at the transcriptional level, we used next-generation sequencing (NGS). Whole kidney cortices were used for bulk sequencing rather than just PTC to gain a broad picture of changes involving perhaps other parts of the nephron, as well. To analyze high-content data set and maximize biological interpretation, we employed Ingenuity Pathway Analysis (IPA), which allows for studying pathway interactions and enrichment. We initially applied stricter, commonly used criteria for differential gene expression analysis: *P* adjusted values (using the Benjamin-Hochberg method for multiple-comparison adjustment) with a cut off of FDR < 0.1. When compared with littermate controls applying such criteria, PT-CrAT^HET^ males had ~2100 genes differentially expressed. Strikingly — and in contrast — in PT-CrAT^HET^ females, the majority of genes did not qualify as differentially expressed to a significant degree. This initial result demonstrates that impairments in mitochondrial substrate efflux cause a much stronger transcriptional response in males compared with females. Relying only on the Benjamin-Hochberg method, however, can result in several false negatives and may eliminate otherwise biologically significant changes (35, 36). Therefore, we reanalyzed our male and female DESeq2 dataset using rather liberal *P* values without adjustment (*P* < 0.1). We found ~4800 genes that were differentially expressed in males and those largely overlapped with the ones found using the FDR < 0.1 criteria. We also found ~1400 genes differentially expressed in PT-CrAT^HET^ females, but the transcriptional responses were again different from those found in males. Figure 5 summarizes the number of differentially expressed genes in males and females and the shared number of genes.

First, we compared the top 10 up- and downregulated genes in PT-CrAT^HET^ males and females. Males had several FA metabolism/oxidation-related genes significantly downregulated (Table 1). Most importantly, *Pdk4* (–22-fold downregulation), which is a regulator of the pyruvate dehydrogenase complex (PDC), and *Cyp4a14* (approximately –18-fold) in connection with FA ω-oxidation in the endoplasmic reticulum and *Hmgcs2* (approximately –11-fold), which encodes a mitochondrial enzyme that catalyzes the first reaction of ketogenesis to provide lipid-derived energy under carbohydrate deprivation, were all significantly impacted. In contrast, females showed significant downregulation of the lncRNA *Fer1l4* (approximately –5-fold) and *Cyp27b1* (approximately –4-fold), which encodes the enzyme 1-α-hydroxylase located in PTC involved in bioactive vitamin D synthesis, among others. When looking at the top 10 upregulated genes, we found for example *Ccdc180*, *Col11a1*, *Gria2*, or *Prdm6* in males in relation to regulation of transcription, renal cell carcinoma, and fibrosis (Table 1). In contrast, we found several genes related to immune response and inflammatory pathways upregulated in females — for example, the chemokine ligand *Cxcl15*, or *Iglv1,* and *Il36A* (Table 1).

**Figure 6:**
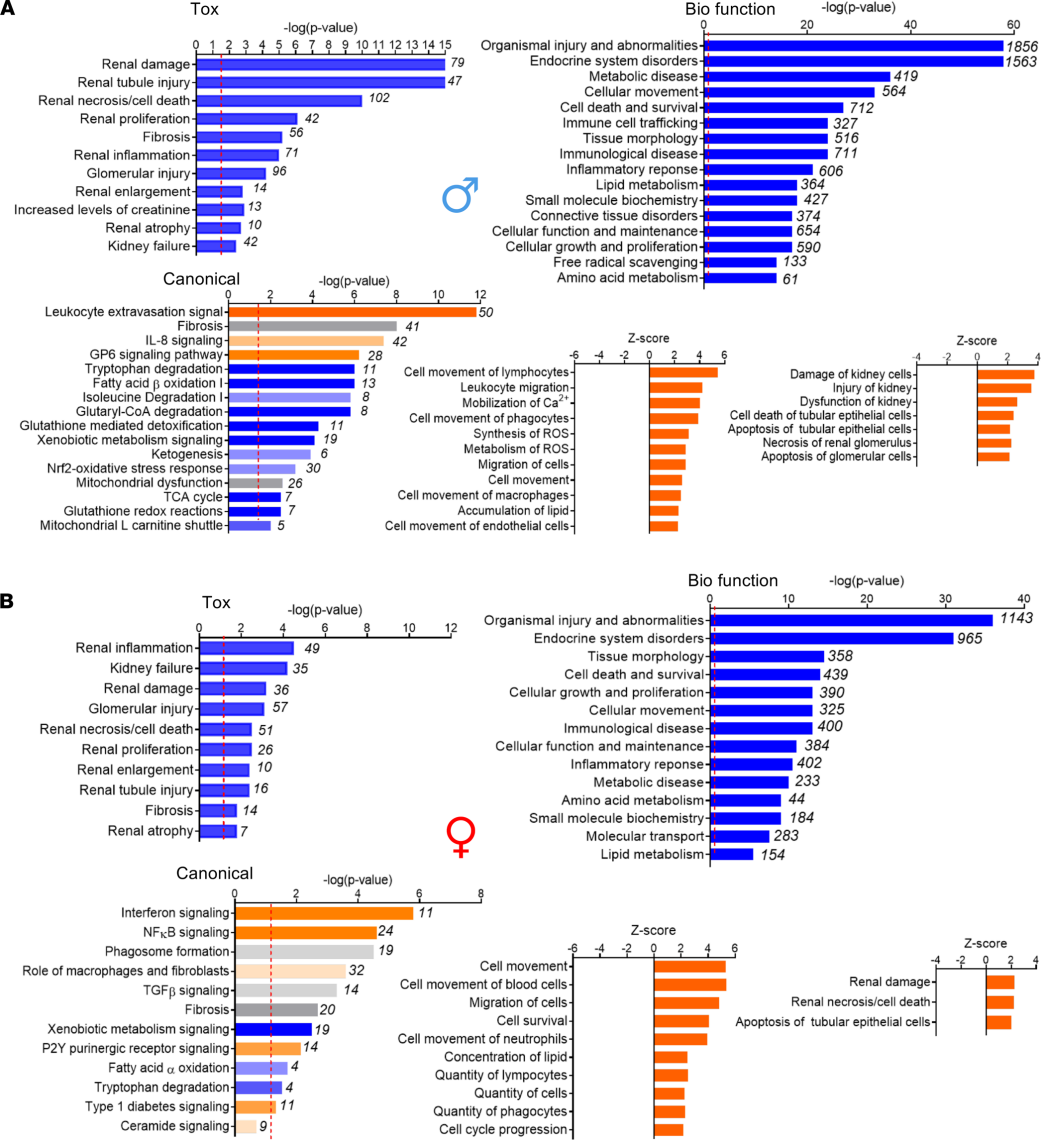
Transcriptomic pathway analyses of male and female kidneys from PT-CrAT^HET^ mice. Transcriptomic pathway analyses of male and female kidneys from PT-CrAT^HET^ mice were performed using Ingenuity Pathway Analysis. The most prominent pathways enriched in disease development regarding toxicology, biological function, canonical pathways, and the activation *Z* scores are shown in males and females. Numbers behind each horizontal bar indicate the number of molecules (genes) found to be differentially expressed in a given pathway in PT-CrAT^HET^ mice. *n* = 3–6 animals/group; genes with a *P* < 0.1 cutoff in differential expression were considered in the analysis. Dashed red line indicates the threshold level.

Next, we performed Core Analysis in IPA to gain insight into specific biological predictions and implicated pathways. With regards to kidney disease development, core analysis predicted renal disease with both tubular and glomerular involvement and fibrosis in both males and females (Figure 6, A and B). Renal inflammation, however, had the highest prominence in females. With regards to biological function, endocrine disorders, metabolic disease, cell death, and lipid and amino acid metabolism and inflammatory responses were among the most significantly enriched (Figure 6, A and B). Lipid and amino acid metabolism pathways and metabolic disease again had a stronger prominence in males versus females, with more genes dysregulated in these pathways in males. When we compared canonical pathways, we found those related to FAO, glutathione-mediated pathways, TCA cycle, ketogenesis, mitochondrial dysfunction, and mitochondrial carnitine shuttle to be the most significantly involved in males (Figure 6A). In females, IFN signaling, NF-κB signaling, macrophage-mediated pathways, and TGF-β signaling were the most prominent ones (Figure 6B), mostly because females had a much weaker transcriptional response in other, metabolism-related pathways. Such differences can also be quickly assessed by comparing the –log(P values) of the core analyses in males versus females, where males had higher readouts when compared with females.

**Figure 7:**
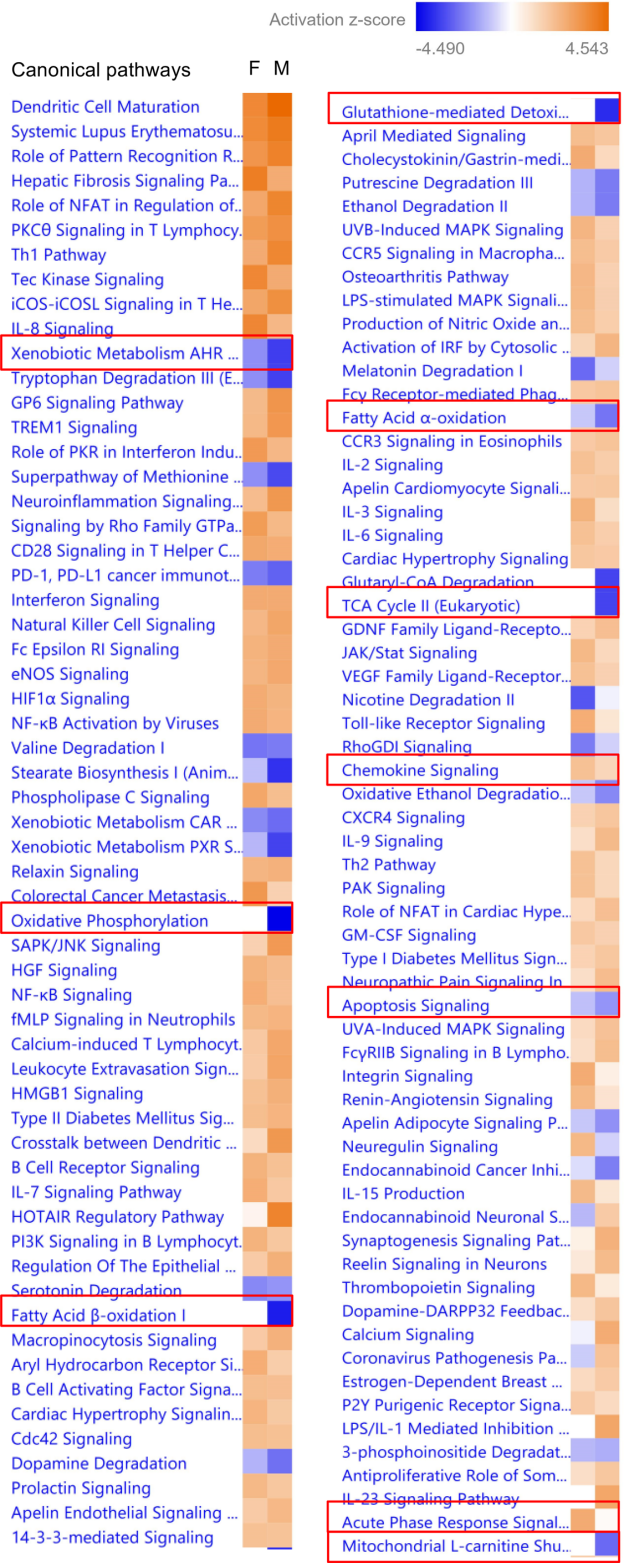
Sex differences in transcriptional pathways in the PT-CrAT^HET^ model. IPA’s Comparison Analysis showing the differences in enrichment of canonical pathways in males versus females based on absolute *Z* scores (inhibited, blue; activated, orange). Pathways with the most relevance to metabolic changes were marked with a red rectangle. *n* = 4–7 animals/group; genes with a *P* < 0.1 cutoff in differential expression were considered in the IPA analysis.

**Figure 8:**
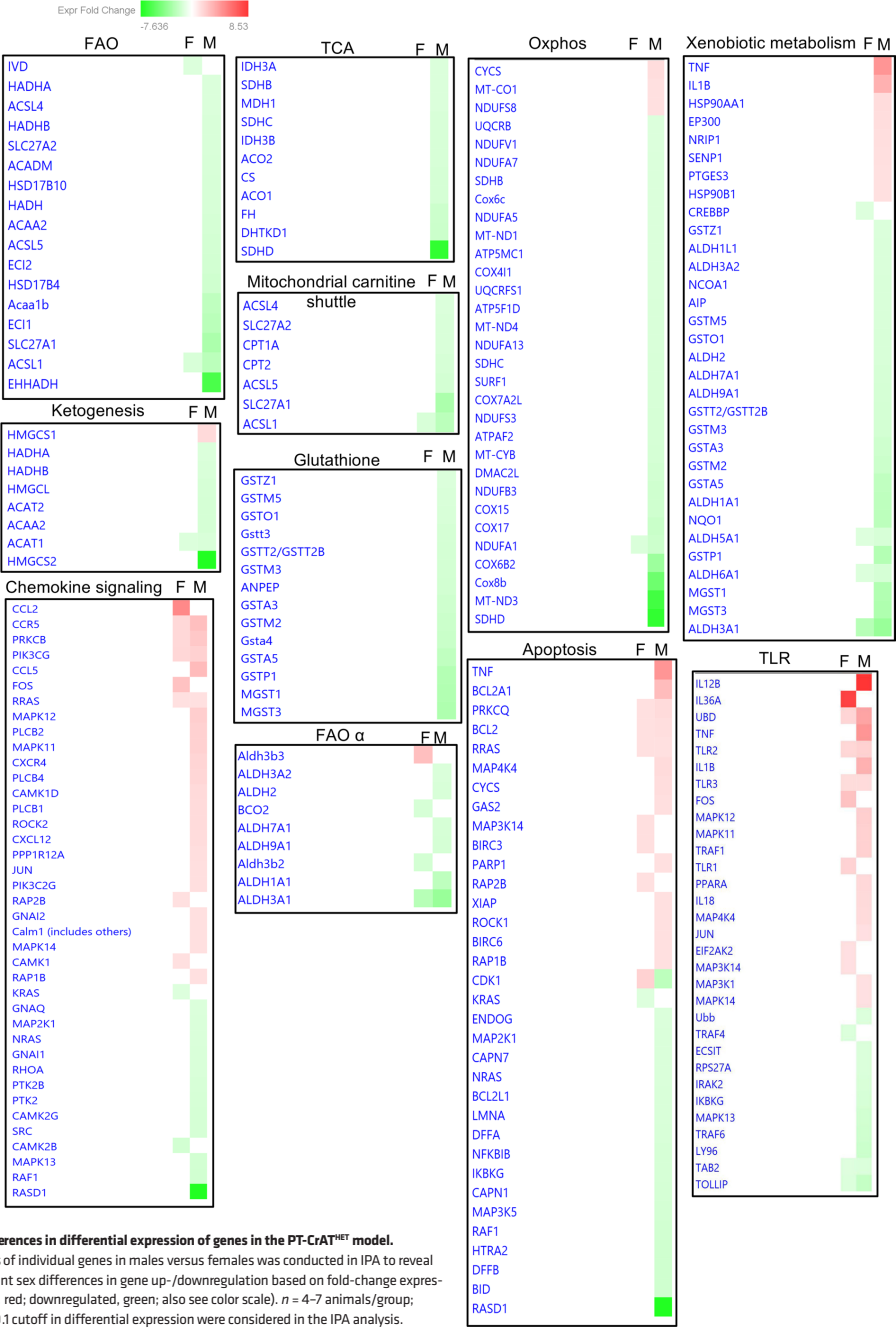
Sex differences in differential expression of genes in the PT-CrAT^HET^ model. Heatmap analysis of individual genes in males versus females was conducted in IPA to reveal the most significant sex differences in gene up-/downregulation based on fold-change expression (upregulated, red; downregulated, green; also see color scale). *n* = 4–7 animals/group; genes with a *P* < 0.1 cutoff in differential expression were considered in the IPA analysis.

**Figure 9:**
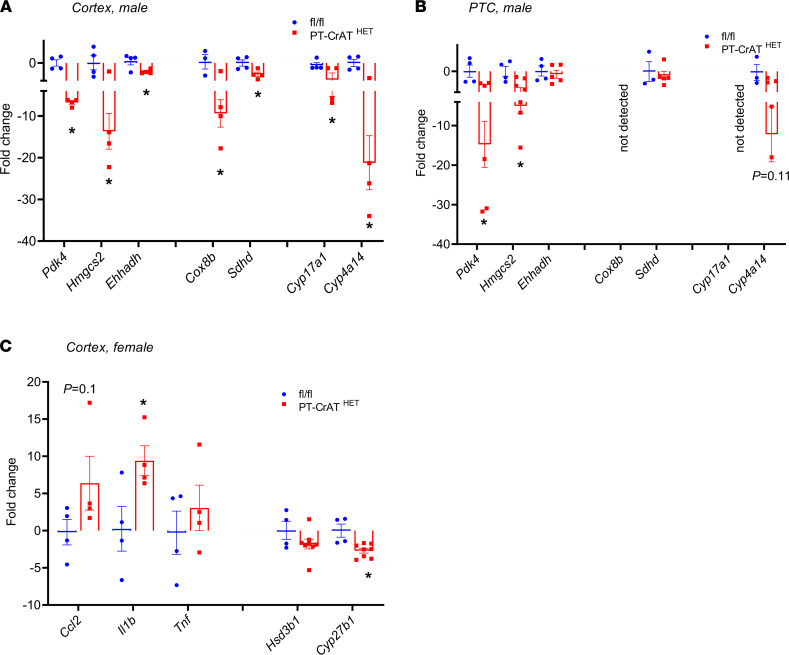
Selected metabolic and inflammatory genes in the PT-CrAT^HET^ model. (**A**–**C**) Some examples of the most relevant genes of metabolic (male) and inflammatory (female) pathways are shown and confirmed using qPCR analysis of male and female cortex (**A** and **C**) and male PTC (**B**). *n* = 4–6 animals/group; *P < 0.05. Data are shown as mean ± SEM; Student’s unpaired *t* test.

Finally, using a gene heatmap comparison analysis, we compared canonical pathways in male and female KOs (Figure 7). We focused on those involved in FA metabolism, FAO, TCA cycle, redox balance, oxidative stress, apoptosis, and inflammation to gain a detailed picture of sexual dimorphism in relation to these pathways. Our most significant findings are detailed in Table 2, Table 3, Table 4, and Table 5, as well as in Figure 7, Figure 8, Figure 9, and Supplemental Figure 4.

### FAO, TCA cycle, and lipid metabolism.

PT-CrAT^HET^ male mice displayed dysregulation of several genes not only in fatty acid β-oxidation, but also α- and ω-oxidation, peroxisomal FAO, defects in mitochondrial carnitine shuttle, and ketogenesis. Importantly, we found strong downregulation of *Pdk4* in males. *Pdk4* encodes a mitochondrial matrix protein that regulates the PDC (37, 38). When Pdk4 is decreased, it leads to dephosphorylation of one of the PDC subunits and a switch to carbohydrate oxidation by increasing the conversion of pyruvate. While *Pdk4* downregulation seems somewhat paradoxical at first, as typically excess lipids would upregulate *Pdk4*, the result is in line with the stronger glycolytic shift observed in males in the respiration experiments. It is possible that *Pdk4* downregulation is a response in PTC to mitigate the ability of excess acetyl-CoA to inhibit use of glucose. With regards to β-oxidation and fatty acid transport/utilization enzymes, we found significant downregulation of several genes encoding such enzymes/proteins in males. Table 2 summarizes the most important findings. In connection with an impairment in FAO, several genes encoding enzymes in the TCA cycle were also downregulated, suggesting a decrease or suppression of TCA cycle capacity. Next, we examined alternative pathways for FAO. ω-Oxidation is an alternative pathway mostly for medium-chain fatty acid metabolism in the ER (39). We found an ~18-fold downregulation of the *Cyp4a14* gene, which encodes cytochrome P450 ω-hydroxylase 4A14. The gene is also an indicator of PPARα activation (40) — which plays a major role in triggering FA utilization and response to lipids — again indicating a major downturn of lipid utilization in PTC with mitochondrial overload in males but not in females. When mitochondrial FAO is impaired, peroxisomal FAO may also serve as an alternative. Intriguingly, we found several genes from peroxisomal FAO and ketogenesis pathways downregulated in males. Finally, genes regulating carnitine shuttle and transport of long-chain fatty acids were significantly downregulated in males. Downregulation of Cpt1a will limit mitochondrial entry of lipids for use as fuel in β-oxidation. FATP2 (encoded by *Slc27a2*) in the kidney is localized to PTC along the apical side (41) and, under pathologic conditions, has been shown to regulate apical nonesterified FA transport, thereby affecting lipotoxicity and apoptosis in PTC. Downregulation of *Slc27a2*, therefore, could be a protective mechanism under mitochondrial overload, in an attempt to prevent further lipotoxic damage. Taken together, these results suggest that even a modest mitochondrial substrate overload in PTC induces a robust transcriptional response in males, leading to an almost complete loss of FAO-related metabolic pathways. Females had a much weaker transcriptional response regarding metabolic pathways.

### Oxphos, xenobiotics (lipid peroxidation), and glutathione mediated pathways.

When we inspected differentially expressed genes encoding/regulating oxidative phosphorylation, we found significant dimorphism again in heterozygous KO males versus females. Table 3 summarizes these findings. Many genes encoding specific subunits of mitochondrial complexes (see Figure 8 OXPHOS subpanel for a full list and sex differences) were downregulated in males. Many of the genes regulating xenobiotic metabolic pathways are interconnected with glutathione metabolism, since glutathione is one of the most important cellular antioxidant defense sources. Males had significant downregulation of genes encoding enzymes in glutathione metabolism pathways — specifically, many subunits of glutathione-S-transferase (see Figure 8 Glutathione detox subpanel for sex differences and Table 3). As these enzymes participate in maintaining the GSH/GSSG balance necessary for removing lipid hydroperoxides (42), we also found several genes encoding enzymes of the aldehyde dehydrogenase family downregulated mostly in male heterozygous KOs. These enzymes remove excess lipid hydroperoxides and, thus, have an important role in maintaining redox balance. Collectively, we can conclude dysregulation of OXPHOS together with an increase in cellular redox imbalance as part of the responses in PT-CrAT^HET^ males, and these results are in line with the observed mitochondrial changes in these animals.

### Chemokine and inflammatory pathways and apoptosis.

In contrast to male PT-CrAT^HET^ KOs, which showed robust responses in fuel metabolism and FAO, responses to mitochondrial overload in females were mostly related to the upregulation of genes related to specific chemokine and inflammatory pathways (Table 4). Specifically, *Il36a* and *Ccl2* showed the strongest response in female KOs. MCP-1 (*Ccl2*) is a known marker of renal inflammation. IL-36A was recently shown to be a reliable marker of progression of tubulointerstitial inflammation in ischemia/reperfusion AKI (43). Males, of course, also showed robust upregulation of inflammatory genes, with a predominantly macrophage-driven inflammatory response related to the upregulation of *Tnf*, *Il1b*, and *Il12b* (Table 4). Both sexes shared some common transcriptional response in relation to chemokines and apoptosis signaling. Notably, some of the significantly upregulated genes included those encoding protein kinase C β and θ, as well as Bcl2. Activation of PKCθ and -β is consistent with a lipotoxic scenario, as these protein kinases can be activated by the secondary messenger diacylglycerol (DAG) (44, 45). Activation of the antiapoptotic Bcl2 is interesting, as it seems to be a response that is attempting to provide protection against deterioration of mitochondrial dynamics and function. Consistent with this observation, males had upregulated genes involved in the initiation of (mito)apoptosis (Table 4).

To further validate our findings, we used quantitative PCR (qPCR) of selected key genes important for metabolic pathways (males) or inflammation (females) and analyzed both cortex and isolated PTC to confirm up- and downregulation, as well as location of transcriptional changes (Figure 9 and Supplemental Figure 4). The results were largely consistent with the NGS analysis in both sexes and showed further details of where metabolic/inflammatory changes occur. Most of the changes of key metabolic genes in males localized to PTC, with the exception of *Cox8b*, which is expressed more in distal tubules, and *Cyp17a1*, which is not a PTC gene. In females, we only used whole cortex, as most of the inflammation-related genes were largely expressed in immune cells but not necessarily PTC. Results in Supplemental Figure 4 are a direct comparison of key metabolic gene expression patterns between males and females and show the weak transcriptional response in those metabolic genes when compared with males. Considered together, these transcriptomic responses are consistent with the previously discussed histopathology and mitochondrial structure data and the functional changes in PTC, but they highlight sex-dependent processes leading to the development of the disease.

### Secondary glomerular injury.

While changes in PTC were numerous as detailed above, when we examined transcriptional changes regarding the secondary glomerular disease, we have not found a particularly strong response. Some notable exceptions are shown in Table 5, such as *Fgf2* and *Ptger1* for males and *Angpt2*, *Nphs2*, and *Synpo* for females regarding glomerular injury. Significant increases in the latter 2 can be interpreted as a compensatory response to the disease. Because of the rather weak transcriptional response, we speculate that the secondary glomerular injury occurs probably due to loss of capillaries and a reduction of glomerular blood flow, or due to paracrine signaling from injured epithelium. Such responses to repeated tubular injury have been described previously (46, 47).

## Discussion

The importance of intact mitochondrial function in preventing many forms of kidney disease has received considerable attention regarding PTC — a renal cell with distinct metabolic features (13, 48–53). However, many questions remain open about metabolic reprogramming of renal cells and alterations in their mitochondrial function, especially in complex metabolic diseases. Our previous work has shown that mitochondrial substrate overload and incomplete FAO in PTC causes CKD (24). For this, we developed a mouse model that exclusively mimics mitochondrial overload (PTC-specific CrAT ablation). Our observations suggest that there is a previously unrecognized role for incomplete mitochondrial FAO in PTC atrophy. The results led us to predict that, in kidney cells with high reliance on mitochondria for energy need like the PTC, mitochondrial substrate overload is a new, important factor leading to CKD.

In this work, our original hypothesis was that we would be able to titrate and estimate the amount of substrate overload in PTC mitochondria by creating a CrAT^HET^ mouse model. Two outcomes were hypothesized: (a) mice will have 50 % of the damage and activation of pathways seen in the homozygous model or perhaps even no evidence of kidney disease, or (b) heterozygous PT-CrAT^HET^ mice will be just as diseased as the homozygous model. Our results show that PT-CrAT^HET^ mice develop kidney injury similarly to their homozygous counterparts. When compared with our previous results (24), the timeline of disease development is similar (~12 months). The severity of pathology and damage was also consistent with what was observed in the homozygous model, in both sexes. Most strikingly, PT-CrAT^HET^ mice not only developed kidney disease, but males showed an almost complete shutdown of FAO pathways, including those in peroxisomes and other alternatives. To our knowledge, it is rarely observed that a 50 % deletion of a metabolic enzyme causes such a striking phenotype and a complete shift in metabolic pathways in the kidney PTC or other tissues. Our results highlight that it is critical for the PTC to have an intact mitochondrial efflux and balance of carbons, acetyl-CoA, and acyl-carnitines. Even a slight disturbance in this process results in kidney disease. As the most important factors for treating CKD/DKD patients currently are controlling blood glucose and blood pressure, results herein point to a new, independent factor in CKD and the importance of considering the specific metabolism of PTC. While our goal herein was to use CrAT deletion as a tool to mimic and achieve overload, it is also noteworthy that CrAT activity itself declines with age and diabetes (18).

**Figure 10:**
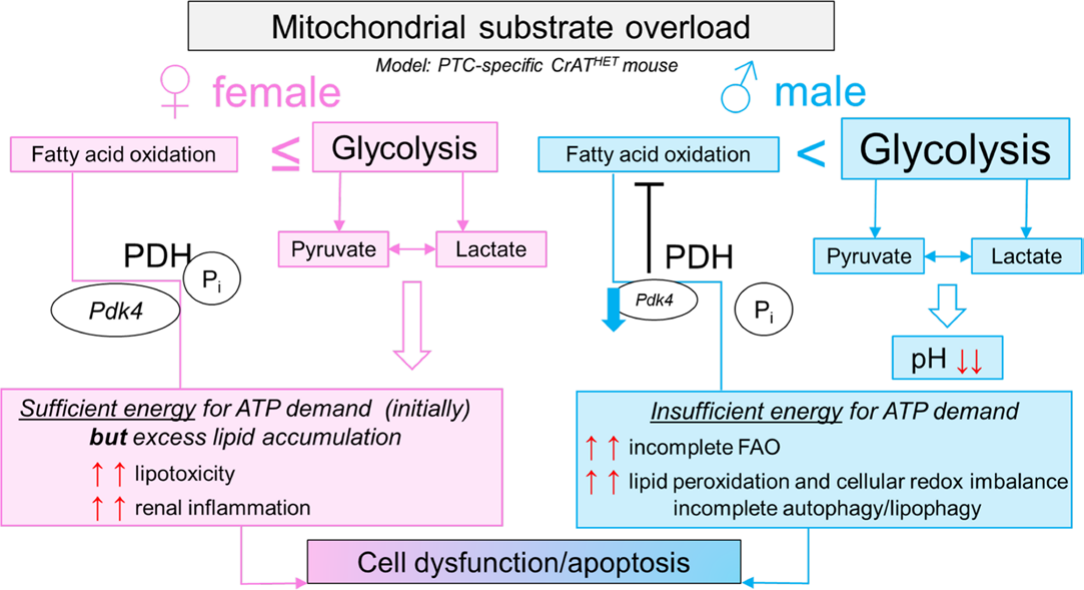
Proposed schematics of mitochondrial substrate overload in PTC.

In addition to the importance of intact mitochondrial substrate efflux, our transcriptomic data set revealed a vast array of differences in dysregulated pathways in males versus females. Several studies examined sex differences in humans and rodent models of both type 1 and type 2 diabetes, where age and timing of the onset of the disease are important factors (26–29, 54, 55). Imbalance in sex hormone levels exacerbates DN (56, 57). The risk for microvascular complications such as end-stage renal disease and proliferative retinopathy is increased in males when age at onset was > 15 years (30). After menopause, the protection seems to weaken in females and the onset of diabetes induces changes in renal gene expression patterns, including those related to mitochondrial metabolism or FAO (*Crot*, *Aldh1a1*, *Hmgcs2*, *Acadm*) (58). The hormonal status affects the progression of diabetic renal damage in animal models, as well (58). Hormone replacement improves renal function and pathology associated with DN or with kidney disease in salt-sensitive hypertension (59–61). In contrast to some extent, others showed that females may be more susceptible to the development of diabetic kidney disease, but they are less likely to develop diabetes (62). While there is a substantial body of work available on sex differences in the kidney, less is known about renal mitochondrial sexual dimorphisms — our recent minireview summarized the most important findings in the field (63). Metabolomic studies on urine biomarkers suggested a distinct signature of mitochondrial dysfunction in DKD in both sexes in patients (64). Importantly to our studies, CKD seemed to progress differently in males and females in the adenine-induced nephropathy and the 5/6 nephrectomy models; however, skeletal muscle mitochondrial function was equally impacted in both (31). Thus, an important finding of our studies is that, in our model, both males and females develop features of CKD by about 12–15 months of age; however, this occurs through different pathways and with some differences regarding the severity of injury. While males show a strong downregulation of *Pdk4*, many of the genes related to medium- and long-chain FA metabolism, genes implicated in peroxisomal or endoplasmic reticulum FAO, and genes related to glutathione metabolism and oxidative stress, these pathways are less prominent in females. In essence, both sexes showed inflammatory responses. However, females exhibited fewer signs of adaptation and remodeling of pathways related to metabolism. Because of the much weaker transcriptional response and adaptation in metabolic pathways, the top scoring pathways in females are activation of inflammatory and chemokine signaling pathways, with the involvement of IL-36, Mcp-1 (*Ccl2*), and similar. The results are in line with the current literature and bring into question the protective role of estrogen and its relationship to FAO. It has been shown that estrogen administration after ovariectomy in rats or in postmenopausal women induces FAO (65–67). It could be surmised, therefore, that female mice have some degree of protection to maintain FAO. PTCs from both males and females show a shift toward glycolysis, though females remain able to use FA-based substrates. It is possible that, in males, the strong *Pdk4* downregulation leads to inhibition of FAO and an increase in glucose oxidation, increasing the conversion of pyruvate to acetyl-CoA, and this results in a “vicious cycle” in mitochondria, which are already experiencing excess levels of acetyl-CoA. Normally, upon mitochondrial acetyl-CoA buildup, the excess would exit in either the form of citrate (formed from oxaloacetate and acetyl-CoA) or acetylcarnitine (by linking acetyl-CoA to carnitine). As acetylcarnitine formation is blocked due to lack of CrAT, there is no alterative pathway to provide relief from mitochondrial substrate and redox imbalance. Females maintain *Pdk4* expression levels, much of their peroxisomal metabolism, and therefore, some level of FAO, probably preserving the ability to slow down acetyl-CoA build-up or to remove excess acetyl-CoA through the citrate shuttle. If, however, excess lipids are stored in lipid droplets (a capacity that is limited in PTC), such ectopic lipid accumulation is a known trigger of chronic inflammation, inflammatory cell infiltration, and secretion of inflammatory cytokines (like IL-18 or C-X-C cytokines) (68, 69). Our data are in line with the literature in a sense that females, in principle, tend to develop more inflammatory and/or autoimmune diseases (26, 70). We can also speculate that mitochondrial processing of fatty acids is important in triggering a proinflammatory response, especially when mitochondria experience overload. This has been recently shown in skeletal muscle using Cpt1b^–/–^ mice (71). Mitochondrial FAO is limited in Cpt1b^–/–^ mice, and this resulted in significant decreases in gene expression of proinflammatory cytokines and chemokines. Preserved mitochondrial FAO in PT-CrAT^HET^ females, thus, could be a reason why their inflammatory response is more pronounced when compared with males. Ultimately, the combination of these changes culminates in the activation of lipoapoptosis and renal damage in males and females (Figure 10).

With regards to the glycolytic shift, we surmise that kidney cells with an impaired efflux of carbons are unable to sense the amount of lipids accumulating around them. It could also be argued that, over time, due to the extent of mitochondrial damage, selective removal of damaged mitochondria — termed as mitophagy — intensifies, as evidenced by the TEM photographs. While this process is important because of the dangers of having damaged mitochondria in the cell, it may also lead to a phenotype in which, due to mitochondrial depletion, PTC attempt preservation of ATP production through enhanced glycolysis. A change in metabolism toward glycolysis resulting in a pH change toward a more acidic environment is a known inducer of autophagy, independently of other molecular mechanisms (72, 73). Autophagy then could certainly be an important mechanism producing the large MLB that we observed in our EM microphotographs in males (74). Premature lamellar bodies fuse with the autophagosome to deliver the cargo to lysosomes where eventual degradation occurs. Disruption in autophagy due to high amounts of lipid droplets exceeding the capacity (75) will result in the accumulation of MLBs. Females seem to be protected from autophagy/lipophagy impairments, although their tubular scores and glomerular tuft size were significantly worse when compared with males, probably indicating the toll of the inflammatory process. We suggest that our observations are also similar to those first described by Otto Warburg in cancer cells in the 1920s and PTC with mitochondrial imbalance of substrate efflux undergoes a Warburg-like transition (76). Perhaps, under lipid overload conditions, PTCs attempt to shift to glycolysis to provide at least some ATP for their high-energy need. This pathway, however, does not seem to be sufficient to meet the high demand of the PTC (that are not primarily glycolytic cells). Alternatively, when enough mitochondrial damage and cellular lipotoxic material accumulates, then — through incomplete autophagy/lipophagy — PTC apoptosis and ultimately kidney disease prevails. Interestingly, a similar glycolytic shift has been shown in polycystic kidney disease (77, 78), where glycolysis accelerates the growth of the cysts while 2-deoxyglucose (2-DG) treatment inhibits glycolysis and slows the growth (79). Recently, an elegant study showed similar glycolytic shifts when *Tfam*, the gene responsible for mitochondrial DNA quality control, was deleted in the kidney epithelium (80). Furthermore, we note that changes during mitochondrial substrate overload also resemble those occurring in acute kidney injury (AKI) and during AKI-to-CKD transition (81–83).

We would like to acknowledge some limitations of our studies. While it is highly informative and provides a robust array of data, transcriptomics has its limits, as it does not provide insight into either protein translation or any posttranslational modification (acetylation of mitochondrial proteins would be a logical one to mention here) that could be part of the picture. However, our structural and functional studies in PTCs are in line with the observed transcript abundance changes, and together, these data strengthen each other and underscore the importance of metabolic shifts in kidney disease development. Unraveling how changes in mitochondrial function, FAO, and glycolytic shifts are intertwined in the PTC under pathologic conditions will undoubtedly serve as a platform for many future studies.

In conclusion, our results highlight 2 observations. First, the integrity of mitochondrial carbon trafficking efflux in PTC is of the utmost importance in preventing kidney disease through the preservation of mitochondrial function. Second, the sexual dimorphism in renal mitochondrial function discovered herein emphasizes the need to tailor our approaches further and consider sex differences in the kidney when possible. If loss of FAO is a “male only” feature, the key targets for future interventions in metabolic disease–related nephropathies may be vastly different in males and females, especially when stimulating FAO is considered as a treatment option.

## Methods

### Animals.

Mice with targeted deletion of CrAT in PTC were generated using Cre-loxP recombination strategy as described previously (24). Homozygous CrATloxP female mice (C57BL/6J background) were originally created by Randall Mynatt at the Pennington Biomedical’s Transgenic Core and bred to male γ-glutamyl-transferase Cre mice (*Tg-[Ggt1-cre]M3Egn/J,* C57BL/6 × SJL background, The Jackson Laboratory). Offspring heterozygous to CrATloxP and Cre^+^ were backcrossed to the CrATloxP, Cre^–^ mice. This cross-produced offspring, of which 50% were PTC-specific CrAT^–/–^ (PT-CrAT) mice and 50 % were CrAT^–/+^ heterozygous KO. Littermate Cre^–^, CrAT^+/+^ mice (fl/fl throughout the manuscript) were used as controls (Supplemental Figure 1). Mice were kept in groups (4/cage) in a room with a 12-hour light/dark cycle and provided food and drinking water ad libitum. All mice were fed a standard rodent chow (Purina LabDiet, 5001). Mice were aged out and monitored for development of kidney disease. At 12–15 months of age, male and female mice were placed into metabolic cages to collect 24-hour urine samples following a period of acclimation. Mice were euthanized and weighed, kidneys were harvested and weighed, and blood was collected for experiments.

### Histology.

Paraffin embedded kidneys were cut into 5 μm cross-sections at the Pennington Biomedical’s Cell Biology and Imaging Core Facility. Sections were mounted on charged SuperFrost slides (Thermo Fisher Scientific) and deparaffinized before staining. Sections were stained with (a) Periodic acid–Schiff (PAS) staining to evaluate glomerular size, mesangial space expansion, sclerosis, protein casts, tubular dilation and atrophy, and potential immune cell infiltration and with (b) trichrome staining for tubulointerstitial fibrosis, casts, and collagen deposits. At least 30 viewing areas per slide were evaluated on each section with a NanoZoomer Digital Pathology Virtual Slide Viewer. The number of sclerotic glomeruli was determined as percentage of glomeruli with pathologic changes including sclerosis, mesangial matrix expansion, and changes in Bowman’s space size per viewing area. Glomeruli were scored on a 0–4 scale, where 0 is a healthy glomerulus with no sclerosis, 1 is 1%–5%, 2 is 5%–25%, 3 is 25%–50%, and 4 is > 50% mesangial expansion and sclerosis. A semiquantitative tubular score was used for evaluating tubular injury, where 0 indicates no lesions, 1 is < 5% of tubular cells with lesions (cast, fibrosis, dilation, atrophy) in the viewing area, 2 is 5%–25 %, 3 is 25%–50%, and 4 is > 50% injured per viewing area. *n* = 6–10 mice per group per sex.

### TEM analysis.

The kidney cortex was separated from medulla and cut into 1 mm^3^ cubes. Kidneys from all experimental groups were fixed in Karnovsky’s fixative. Samples were analyzed using a Jeol 1010 Transmission Electron Microscope at the Medical University of South Carolina. Several (*n* = 60–80) microphotographs were taken from each sample at various magnifications (×10,000 to ×40,000) and evaluated for: mitochondrial swelling, fragmentation, presence of disorganized cristae, lipid droplets, presence of autolysosomes (signs of autophagic/mitophagic activity), presence of MLB, and podocyte foot effacement. We compared at least 30–40 viewing areas of the same magnification per sample for analyses. Mitochondrial circularity and matrix density were measured using the ImageJ software (NIH). Total counts were compared using 2-tailed Student’s *t* test, and significance was considered at *P* < 0.05.

### Isolation of mRNA.

For RNA isolation, kidney cortices were harvested into 500 μL TriZol reagent and processed the same day when possible or were stored in the TriZol solution at –80°C until processing. Total RNA was isolated from kidney cortices using the RNeasy Mini Kit (QIAGEN). The RNA isolation was not restricted to PT only, and the whole kidney cortex was used to see the overall effect of PT-specific overload on the cortex. Concentration and integrity of the RNA were measured using a NanoDrop ND-1000 spectrophotometer and an Agilent 2100 Bioanalyzer.

### NGS and analysis.

For NGS we used the QuantSeq 3′ mRNA-Seq Library Prep Kit FWD for Illumina platform (Lexogen Gmbh). Sequencing libraries from the samples were generated from 200 ng/μL total RNA as input material. Differential expression between control and PT-CrAT^HET^ kidney samples was analyzed using the R/Bioconductor program DESeq2. Considering differential gene expression, 2 cutoffs were applied. First, adjustments for multiple testing were performed using the Benjamin-Hochberg procedure (with a cutoff of *P* adjusted FDR < 0.1) as a standard procedure. After discovering the transcriptional differences between males and females, we also compared genes and pathways based on *P* values, and genes were considered differentially expressed with *P* < 0.1 in each experiment.

The majority of the overrepresentation and enrichment analysis was conducted using Qiagen’s IPA software (Ingenuity Systems Inc.). The top 10 up- and downregulated genes (determined by fold-change comparison) were compared using the DESeq2 analysis table and IPA software. The most prominent biological function, toxicology, and canonical pathways were selected from the IPA software Core Analysis to predict effected biology with relevance to kidney disease. Sex differences in canonical pathways and differently expressed genes between males and females were compared based on the activation *Z* score and gene heatmap analysis from IPA’s “Comparison Analysis” function. Cluster analysis was done using the normalized counts from the DESeq2 tables by Stanford University’s open access Cluster Analysis 3.0 and Tree View software. NGS data with normalized counts are made available from GEO under accession no. GSE171271.

### Real-time PCR.

Total RNA was extracted as described above. Complementary DNA was synthesized by reverse transcription using iScript (Bio-Rad). Gene expression was measured by quantitative PCR (Applied Biosystems) using TaqMan Assay system and primers (Thermo Fisher Scientific). *Gapdh* was used to normalize the expression of target genes. All mouse primers were purchased as part of the TaqMan Assay system from Thermo Fisher Scientific and included in Supplemental Table 1.

### Serum creatinine.

Creatinine levels in serum were measured using a mouse Creatinine kit (Cayman Chemicals). Western blotting was done using standard methods of gel electrophoresis, and antibodies are detailed in Supplemental Table 2.

### XF24e extracellular flux analysis.

Kidneys from PT-CrAT^HET^ and littermate control mice were harvested and placed into ice cold Krebs-Henseleit buffer for primary PTC isolation. PTCs were isolated using a collagenase digestion (1 % collagenase I at 37°C for 30 minutes in DMEM buffer) and sequential sieving method originally described by Terryn et al. (84) and later modified by our laboratory (85). Briefly, the digested suspension was sieved through 2 nylon sieves with pore size of 250 μm and 75 μm (Gilson Inc.). The top of the 75 μm sieve was washed with DMEM (washing the sieve in reverse direction) to collect 80–100 μm PT fragments. Protein content was measured from the pellet and equalized then PTC segments were plated onto 24-well XF24e Seahorse culture plates. PTC were left undisturbed at 37°C for 2 hours in serum-free media to deplete endogenous substrates so that they readily accept the substrates provided in the measurements. Mitochondrial oxygen consumption was measured in cells respirating on 5 mM pyruvate or 150 μM palmitate conjugated to BSA using an Agilent Seahorse XF24e Extracellular Flux Analyzer. To determine respiratory parameters (basal, ATP-linked, maximal, reserve and leak respiration, nonmitochondrial respiration), sequential injections of 2 μM oligomycin, 1 μM FCCP, and 1.5 μg/mL antimycin A were used in the ports. For fatty acid based respiratory parameters, 50 μM ETX and 2 μM oligomycin were used. To test glycolysis, glycolytic capacity and glycolytic reserve, 10 mM glucose, 2 μM oligomycin, and 50 mM 2-DG were injected sequentially in a second experiment, and changes in pH levels were monitored as ECAR. All parameters were calculated using the Agilent Wave software. *n* = 8 mice/group total, *n* = 10 technical replicates per each experiment.

### Statistics.

Statistical analyses were conducted using GraphPad Prism 8.0 statistical analysis. Two-way ANOVA (Bonferroni’s post hoc analysis) was used to compare sex differences of parameters between strains. In case of data sets with unequal variances, Kruskal-Wallis test was performed. Two-tailed Student’s unpaired *t* test assuming unequal variances or Mann-Whitney *U* test for data sets with non-Gaussian distribution were used to compare control and PT-CrAT^HET^ animals. Differences were considered statistically significant when *P* < 0.05.

### Study approval.

All animal experiments were approved by Pennington Biomedical Research Center’s IACUC and were performed at the Association for Accreditation of Laboratory Animal Care–accredited (AAALAC-accredited) facility.

## Author contributions

KS designed the research. AM, KMC, CK, MEM, SJB, EB, and TD performed experiments. DVI and KNM performed the TEM analysis. RCN contributed concepts and discussion of hypotheses. CK provided expertise with transcriptomic and bioinformatic analysis. AM, KMC, and KD analyzed data and contributed to figures. KS wrote the manuscript and discussed outcomes and interpretations with RCN and DVI. All authors discussed and reviewed the final version of the manuscript.

## Supplementary Material

Supplemental data

## Figures and Tables

**Table 1:**
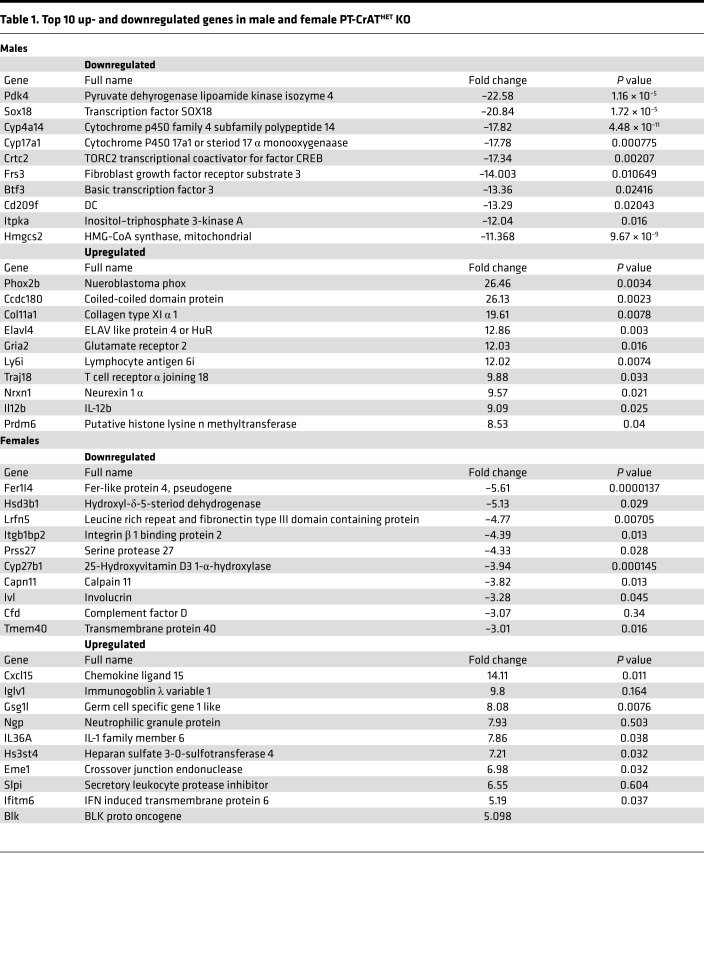
Top 10 up- and downregulated genes in male and female PT-CrAT^HET^ KO

**Table 2:**
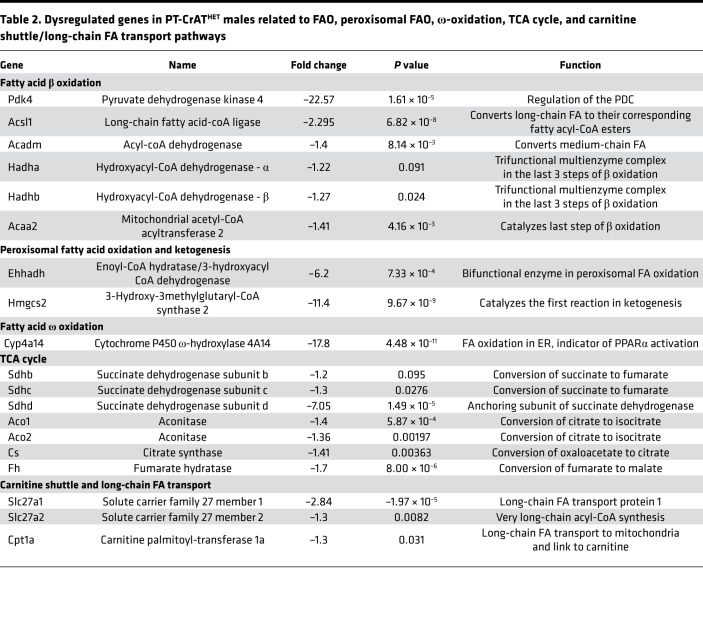
Dysregulated genes in PT-CrAT^HET^ males related to FAO, peroxisomal FAO, ω-oxidation, TCA cycle, and carnitine shuttle/long-chain FA transport pathways

**Table 3:**
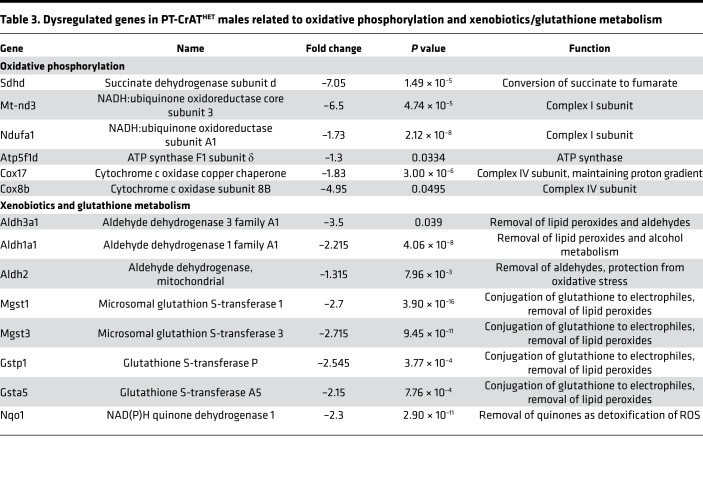
Dysregulated genes in PT-CrAT^HET^ males related to oxidative phosphorylation and xenobiotics/glutathione metabolism

**Table 4:**
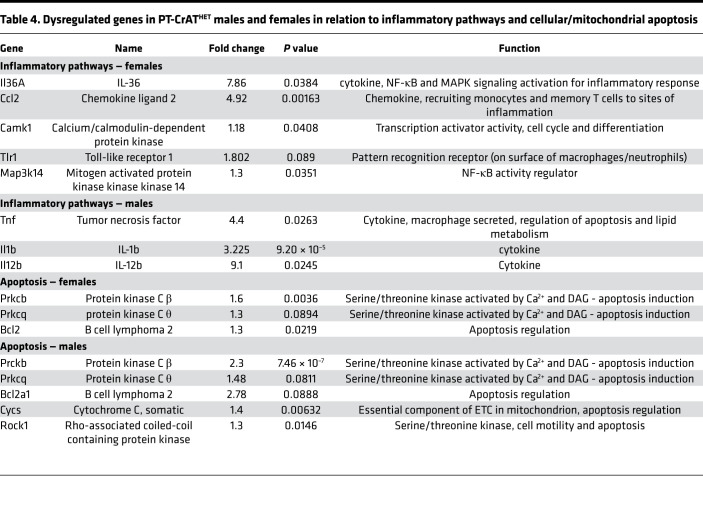
Dysregulated genes in PT-CrAT^HET^ males and females in relation to inflammatory pathways and cellular/mitochondrial apoptosis

**Table 5:**
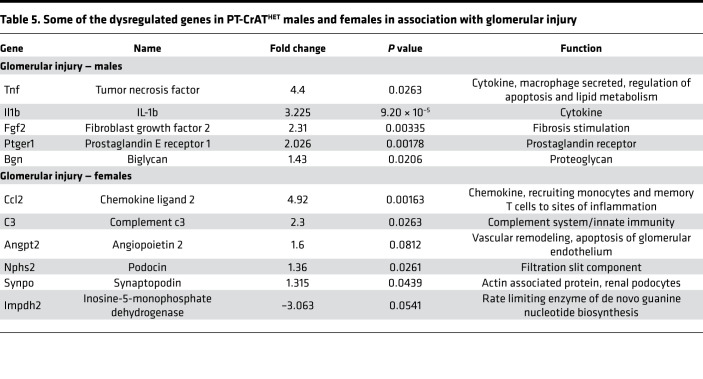
Some of the dysregulated genes in PT-CrAT^HET^ males and females in association with glomerular injury

## Version 1 03/01/2022

In-Press Preview

## Version 2 04/08/2022

Electronic publication
