# Supplementary material for: Intact mitochondrial substrate efflux is essential for prevention of tubular injury in a sex-dependent manner
Source: JCI Insight. 2022 Apr 8;7(7):e150696. doi: 10.1172/jci.insight.150696 (PMC9057616; doi:10.1172/jci.insight.150696)
Supplement: Supplemental data [file jciinsight-7-150696-s159.pdf]

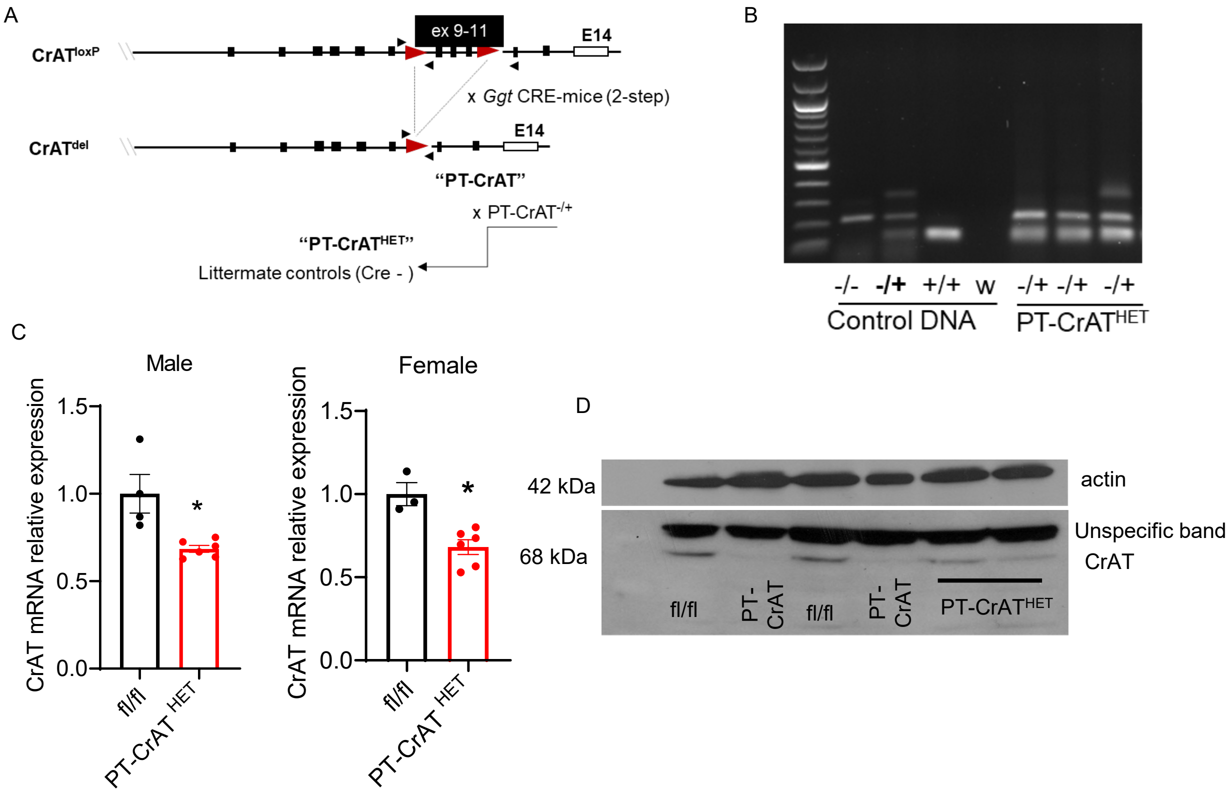

Figure S1. Breeding strategy and generation of PT-CrAT^{HET} cohorts. (A) Mice with floxed CrAT alleles (exons 9-11 deleted) were mated with Ggt CRE mice to create homozygous PT-CrAT mice in a two-step breeding strategy. (B) Polymerase chain reaction (PCR) and genotyping (tail DNA) of PT-CrAT^{HET} mice. -/- : full knockout, -/+: heterozygous knockout, +/+: wild type, w: water as controls. (C) CrAT mRNA expression levels in the kidney cortex of male and female mice. (D) Western blot analysis of CrAT protein levels in isolated PTC (fl/fl controls and both full knockouts and heterozygous knockouts are shown for validation). N=3-6/group, mean ± SEM, * P<0.05, Student's *t*-test.

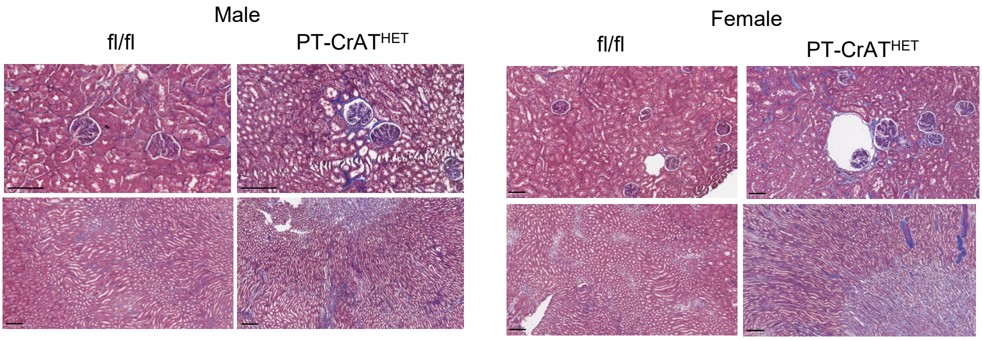

Figure S2. Masson's TriChrome staining showing collagen deposits and fibrosis in both cortex and medulla of PT-CrAT^{HET} mice at 12-15 months of age. N=5-7/group, representative from n=~30-40 pictures/mouse kidney at the same magnification, scale bar = 200 μm.

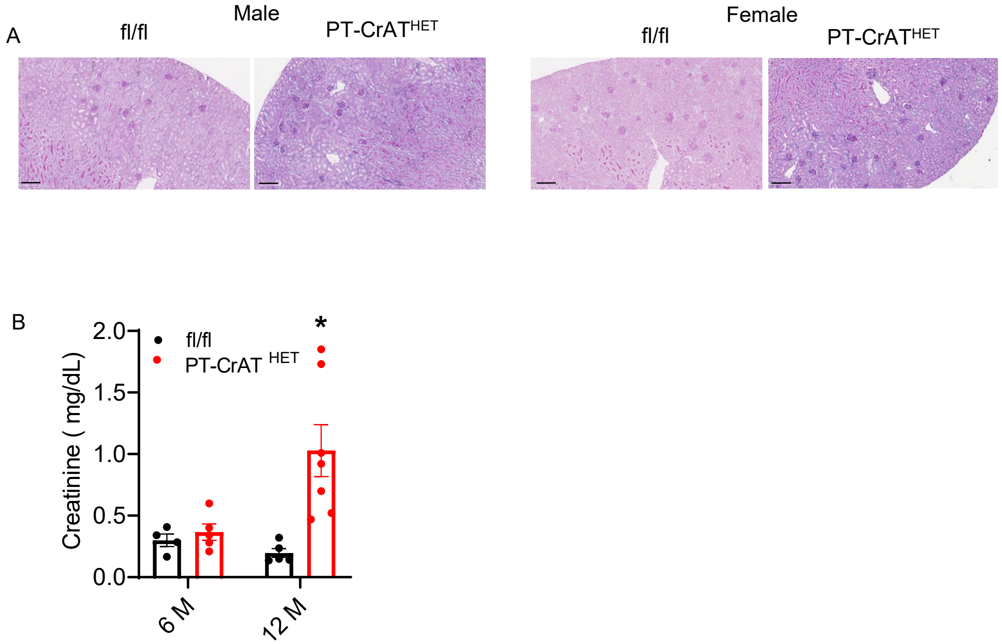

Figure S3. Disease development in PT-CrAT^{HET} mice. (A) PAS staining in younger, 6 month old male and female mice with normal histology. (B) Serum creatinine levels at 6 and 12 months of age (males). N=4-7/group, mean ± SEM, * $P<0.05$, Two-way ANOVA/Bonferroni post hoc test. Scale bar = 200 µm.

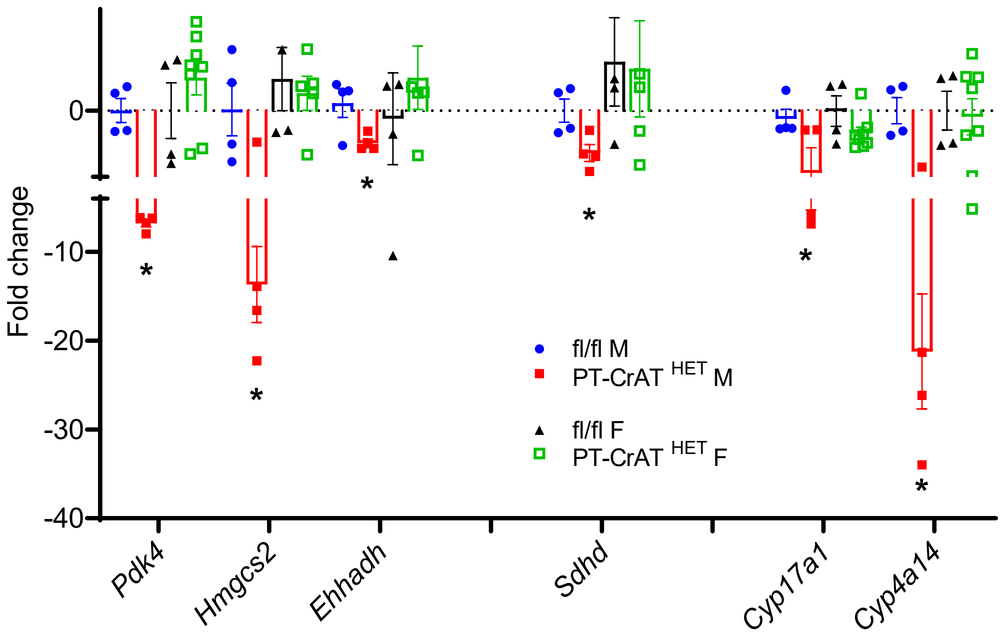

Figure S4 Comparison of expression of selected metabolic genes in male vs female PT-CrAT^{HET} mice. qPCR analysis was performed as a secondary method to confirm some of the top metabolic gene expression differences shown in Fig. 5B. N=4-6/group, mean ± SEM,
* *P*<0.05. Two-way ANOVA, Bonferroni's post-hoc test.

Gene	ThermoFisher primer
Pdk4	Taqman Gene Expression Assay Primer - Pdk4(Mm01166879_m1)
Sdhd	Taqman Gene Expression Assay Primer - Sdhd (Mm00546511_m1)
Ehhadh	Taqman Gene Expression Assay Primer - Ehhadh (Mm00619685_m1)
Aldh3a1	Taqman Gene Expression Assay Primer – Aldh3a1 (Mm00839312_m1)
Hmgcs2	Taqman Gene Expression Assay Primer –Hmgcs2 (Mm00550050_m1)
Cox8b	Taqman Gene Expression Assay Primer – Cox8b (Mm00432648_m1)
Ccl2	Taqman Gene Expression Assay Primer – Ccl2 (Mm00441242_m1)
Tnf	Taqman Gene Expression Assay Primer – TnF (Mm00434228_m1)
Il1b	Taqman Gene Expression Assay Primer – Il1b (Mm00434228_m1)
Hsd3b1	Taqman Gene Expression Assay Primer – Hsd3b1 (Mm00476184_g1)
Cyp27b1	Taqman Gene Expression Assay Primer – Cyp27b1 (Mm01165918_g1)
Cyp17a1	Taqman Gene Expression Assay Primer - Cyp17a1 (Mm00484040_m1)
Cyp4a14	Taqman Gene Expression Assay Primer - Cyp4a14 (Mm00484135_m1)
Gapdh	Taqman Gene Expression Assay Primer - Gapdh (Mm99999915_g1)

Suppl. Table 1. List of ThermoFisher primers used.

Antibody	Manufacturer	Cat. No.	Ab dilution
β-actin	Abcam	AB8226	1:10 000
LC3I/II	Cell Signaling Technology	4108S	1:500
CrAT	Proteintech	15170-1-AP	1:250

Suppl. Table 2. List of antibodies used.
